# Supplementary material for: Analysis of Procollagen C-Proteinase Enhancer-1/Glycosaminoglycan Binding Sites and of the Potential Role of Calcium Ions in the Interaction
Source: Int J Mol Sci. 2019 Oct 10;20(20):5021. doi: 10.3390/ijms20205021 (PMC6829435; doi:10.3390/ijms20205021)
Supplement: Supplementary file 1 [file ijms-20-05021-s001.pdf]

Supplementary Materials

Analysis of procollagen C-proteinase enhancer-1/glycosaminoglycan binding sites and of the potential role of calcium ions in the interaction

Jan Potthoff, Krzysztof K. Bojarski, Gergely Kohut, Agnieszka G. Lipska, Adam Liwo, Efrat Kessler, Sylvie Ricard-Blum, and Sergey A. Samsonov

Table S1. Molecular docking MD-based analysis summary for CUB1-CUB2 domain/GAG interaction.

GAG	[1]m, ε	[2]<br>\#	[3]<br>Size	[4]ΔG,<br>kcal/mol	[5]$Top_{MM-GBSA}$ 10 residues for GAG binding	[6]Polarity
CS6,<br>dp4	3, 3	1	17	-20.2±12.1	A64, R66, R85, Y187, R189, R206, K209, F210, C211, P216	16/1
		2	9	-24.1±6.7<br>-50.6±5.4	A64, R66, R82, L83, G84, R85, Y187, R189, R206, V231	8/1
		3	5	-4.8±6.0<br>-27.9±6.7	R82, G84, R85, P92, A93, Y187, R189, S204, R206, K209	5/0
		4	5	-22.2±7.2<br>-30.7±11.5	A64, R66, R82, R85, F86, F90, Y187, R189, R206, K209	4/1
CS6,<br>dp6	3, 3	1	17	-28.8±7.8<br>-21.6±6.5<br>-13.6±8.4<br>-31.0±11.0	H62, R66, R82, R85, Y187, C188, R189, R206, K209, F210	17/0
DS,<br>dp6	3, 3	1	4	-15.4±9.8	R66, Y67, R82, R85, R105, R189, R205, R206, G208, K209	2/2
		2	3	-34.9±8.2<br>-25.7±9.6	A64, R66, R82, R85, R105, T186, C188, R189, R206, K209	2/1
HP,<br>dp2	1.2, 4	1	9	-16.0±10.1<br>-15.9±4.9<br>-21.3±6.6	R66, N81, R82, G84, R85, R105, F180, L180, A199, D202	9/0
		2	7	-22.8±7.0<br>-10.4±8.3<br>-37.9±14.7	A64, R66, R82, G84, R85, R105, F180, L180, A199, D202	7/0
		3	5	-16.9±11.4<br>-8.2±14.0<br>-13.9±7.5	A64, R66, R82, R85, F86, C87, F180, L180, A199, D202	5/0
HP,<br>dp4	2.1, 2	1	5	-18.9±6.4<br>-14.6±8.2<br>-14.0±8.5	A64, R66, R82, G84, R85, F180, L180, G198, A199, D202	5/0
		2	3	-28.1±9.1<br>-37.7±13.7<br>-41.1±12.6	R66, N81, R82, G84, R85, R105, L180, G198, A199, D202	3/0
		3	3	-6.3±13.1<br>-7.4±15.4	R66, R82, R85, R105, F180, L180, G198, A199, S201, D202	3/0

				-34.9±10.9		
HP, dp6	2.7, 2	1	7	22.1±9.8<br>-27.5±15.6<br>-9.2±12.5	R66, R82, R85, R105, F180, L180, A199, S201, D202, D203	7/0
		2	6	-5.7±15.4<br>4.2±11.7<br>-20.6±9.7	R66, N81, R82, R85, R105, F180, L180, G198, A199, D202	6/0
		3	3	-22.2±26.1<br>-27.3±14.0<br>-16.4±11.1	A64, R66, R82, R85, R105, F180, L180, G198, A199, D202	2/1

[1] DBSCAN parameters *m,* the minimal neighborhood size and ε, neighborhood search radius [33]; [2] Cluster number; [3] Cluster size; [4] Free energy of binding obtained by MM-GBSA; [5] Residues identified in the top 10 for binding according to MM-GBSA calculations pro cluster. [6] The polarity of a GAG binding pose was defined as its preferred orientation in relation to the reducing and non-reducing end.

Table S2. Similarity of GAG binding poses for the CUB1-CUB2 domains as of the number of common amino acid residues identified in the top 10 for binding according to MM-GBSA calculations per cluster.

GAG	CS6, dp4				CS6, dp6	DS, dp6		HP, dp2			HP, dp4			HP, dp6		
CS6, dp4	10	6	5	7	7	5	6	6	7	7	7	5	6	7	6	7
	6	10	6	7	6	5	6	7	8	7	8	6	6	6	6	7
	5	6	10	6	6	5	5	7	7	6	7	6	6	6	6	6
	7	7	6	10	7	6	7	7	8	9	8	6	7	7	7	8
CS6, dp6	7	6	6	7	10	6	7	7	7	7	7	6	7	8	7	7
DS, dp6	5	5	5	6	6	10	7	7	7	6	7	8	9	8	8	8
	6	6	5	7	7	7	10	7	8	7	7	7	7	7	7	7
HP, dp2	6	7	7	7	7	7	7	10	9	7	8	9	8	8	9	8
	7	8	7	8	7	7	8	9	10	8	9	8	8	8	8	9
	7	7	6	9	7	6	7	7	8	10	8	6	7	7	7	8
HP, dp4	7	8	7	8	7	7	7	8	9	8	10	8	8	7	8	9
	5	6	6	6	6	8	7	9	8	6	8	10	8	7	9	8
	6	6	6	7	7	9	7	8	8	7	8	8	10	9	9	9
HP, dp6	7	6	6	7	8	8	7	8	8	7	7	7	9	10	8	8
	6	6	6	7	7	8	7	9	8	7	8	9	9	8	10	9
	7	7	6	8	7	8	8	8	9	8	9	8	9	8	9	10

Each line/column in front/below each GAG reflects a separate cluster, for which average values were taken into account.

Table S3. Similarity of GAG binding poses for the CUB1-CUB2 domains as of the number of common amino acid residues identified in the top 10 for binding according to MM-GBSA calculations per GAG.

GAG	CS6, dp4	CS6, dp6	DS, dp6	HP, dp2	HP, dp4	HP, dp6
CS6, dp4	10	7	7	9	9	7
CS6, dp6	7	10	7	7	7	7
DS, dp6	7	7	10	6	8	7
HP, dp2	9	7	6	10	8	8
HP, dp4	9	7	8	8	10	8
HP, dp6	7	7	7	8	8	10

Table S4. Molecular docking MD-based analysis summary for PCPE-1 UNRES models/GAG interaction.

Structure	[1]m,ε	[2]#	[3]Size	[4]ΔG, kcal/mol	[5]Top$_{MM\text{-}GBSA}$ 10 residues for GAG binding	[6]Polarity
Model 1<br>HP, dp6	2, 2.5	1	7	-90.3 ± 13.7<br>-80.2 ± 16.9<br>-80.8 ± 23.0	R275, R323, K287, R288, R324, K320, K271, L273, K204, K393	7/0
		2	4	-87.3 ± 20.3<br>-70.1 ± 13.0<br>-78.8 ± 10.6	R323, R288, R275, K287, K279, K320, R324, K271, L273, G276	4/0
		3	3	-100.7 ± 17.4<br>-86.7 ± 20.5<br>-102.2 ± 21.3	R323, R288, K287, R324, R275, K320, K279, R422, K393, K271	3/0
Model 2<br>HP, dp6	2, 3.0	1	12	-58.1 ± 12.6<br>-59.3 ± 15.3<br>-82.5 ± 23.4	K394, R348, K393, K295, K293, R324, R422, K299, K305, P298	11/1
		2	4	-54.9 ± 12.9<br>-10.1 ± 8.7<br>-60.0 ± 14.3	R288, K295, K293, K344, V294, K287, G289, K394, K204, K271	3/1
		3	3	-66.7 ± 15.2<br>-28.9 ± 8.7<br>-46.1 ± 12.7	K295, R288, K293, R348, V294, K394, K344, K287, K393, K299	3/0
Model 3<br>HP, dp6	2, 3.2	1	14	-63.1 ± 11.6<br>-51.2 ± 12.6<br>-67.6 ± 11.1	R422, K393, R324, K394, K344, H421, R348, N424, V396, R323,	12/2
		2	8	-84.0 ± 10.9<br>-73.6 ± 11.4<br>-90.9 ± 13.2	K393, K394, R348, R422, M346, K344, H421, R324, G395, S345	7/1
		3	3	-61.5 ± 23.1<br>-58.3 ± 9.2<br>-94.1 ± 15.6	K393, K394, R348, K344, R422, G395, K387, K320, V396, Q388	2/1

Table S5. Molecular docking MD-based analysis summary for NTR domain/Ca^{2+}/GAG system.

GAG	[1] m, ε	[2] #	[3] Size	[4]ΔG, kcal/mol	[5]Top$_{MM\text{-}GBSA}$ 10 residues for GAG binding	Polarity
HP, dp2	4, 0.85	1	19	-46.2 ± 7.6<br>-37.5 ± 8.0<br>-39.0 ± 12.8	R323, C389, Q321, C322, P390, P391, R324, P414, Q388, K320	19/0
		2	10	-42.0 ± 8.7<br>-20.6 ± 5.9<br>-33.2 ± 9.6	R323, R348, K393, R324, K394, K387, K320, Q388, Q321, M346	10/0
		3	6	-19.6 ± 5.5<br>-12.7 ± 9.7<br>-14.8 ± 5.8	K436, K365, K434, C437, R435, G367, T366, Q440, R324, R408	6/0
HP, dp4	2, 2.5	1	17	-46.7 ± 7.8<br>-45.1 ± 10.6<br>-52.1 ± 11.4	R324, R323, R422, K393, Q321, L420, V419, P391, K320, K387	14/3
		2	6	-44.9 ± 14.3<br>-49.5 ± 7.4<br>-39.1 ± 7.9	R324, R323, Q321, K393, R422, K320, V419, P391, L420, C322	4/2
		3	3	-45.5 ± 7.6<br>-61.1 ± 8.2<br>-62.1 ± 6.7	R323, K320, Q321, R422, V419, C322, T327, R324, T325, K393	3/0
HP, dp6	2, 2.5	1	3	-46.9 ± 11.6<br>-68.0 ± 18.3<br>-50.6 ± 12.7	R348, K393, K394, K387, R324, K320, R323, Q388, P391, V347	3/0
		2	3	-72.8 ± 9.0<br>-88.6 ± 12.4<br>-44.5 ± 6.6	K393, R323, K394, R324, R348, K387, R422, K320, P391, L420	2/1
		3	3	-42.2 ± 11.2<br>-31.5 ±11.3<br>-18.8 ± 10.6	K436, K365, R435, G368, K434, G367, L369, S336, C437, T366	3/0

[1] DBSCAN parameters *m*, the minimal neighborhood size and ε, neighborhood search radius [33]; [2] Cluster number; [3] Cluster size; [4] Free energy of binding obtained by MM-GBSA; [5] Residues identified in the top 10 for binding according to MM-GBSA calculations pro cluster.

Table S6. Molecular docking MD-based analysis summary for CUB1-CUB2 domain/Ca^{2+}/GAG interaction.

GAG	[1] m, ε	[2] #	[3] Size	[4] ΔG, kcal/mol	[5] $Top_{MM\text{-}GBSA}$ 10 residues for GAG binding	Polarity
HP, dp2	4, 1	1	10	-14.0 ± 10.5<br>-46.9 ± 6.3<br>-4.6 ± 11.1	R231, K234, R107, L120, Q254, R214, V256, L108, V121, G109	10/0
		2	6	-16.1 ± 8.4<br>-28.6 ± 10.4<br>-20.2 ± 8.4	R107, R231, K234, R110, Q106, G109, R214, Y212, G233, R130	6/0
		3	5	-21.1 ± 7.0<br>-27.0 ± 7.8<br>-15.9 ± 13.5	K234, R107, Q106, Y212, G233, F221, L232, R231, L108, S219	5/0
HP, dp4	3, 2	1	7	-13.6 ± 9.2<br>1.4 ± 9.1<br>6.9 ± 10.0	R231, K234, R107, Q106, R91, G233, R110, R214, R130, R230	7/0
		2	6	-42.1 ± 14.2<br>-31.1 ± 9.5<br>-40.4 ± 11.6	R231, R130, R110, R91, K234, Q106, Y212, R107, R214, G233	6/0
		3	5	-14.4 ± 10.9<br>-0.4 ± 5.6<br>-29.9 ± 8.8	K234, R231, R107, R110, T211, F235, Y212, G233, Q106, C236	5/0
HP, dp6	2, 2.45	1	17	-14.4 ± 17.0<br>-31.9 ± 9.4<br>-21.1 ± 18.7	R130, R91, R231, K234, Y212, R214, R107, R230, Q106, R110	17/0
		2	9	-31.3 ± 11.1<br>-31.2 ± 12.2<br>-41.4 ± 7.8	R110, K234, R130, R231, Y212, R214, G109, R107, R91, K61	9/0
		3	3	-51.3 ± 9.9<br>-42.2 ± 19.4<br>-29.0 ± 11.7	R91, R231, K234, Q106, R107, R214, K61, G109, R110, R230	3/0

[1] DBSCAN parameters *m*, the minimal neighborhood size and ε, neighborhood search radius [33]; [2] Cluster number; [3] Cluster size; [4] Free energy of binding obtained by MM-GBSA; [5] Residues identified in the top 10 for binding according to MM-GBSA calculations pro cluster.

Table S7. Molecular docking MD-based analysis summary for PCPE-1 UNRES models/Ca^{2+}/GAG interaction.

Structure	[1]m,ε	[2]#	[3]Size	[4]ΔG, kcal/mol	[5]Top$_{MM-GBSA}$ 10 residues for GAG binding	[6]Polarity
MODEL 1 HP, dp6	2, 3.0	1	6	-78.0 ± 20.2<br>-70.2 ± 10.0<br>-77.6 ± 15.7	K393, R324, R323, K394, R288, K287, K320, M392, C322, K271	3/3
		2	3	-99.1 ± 20.0<br>-94.0 ± 19.8<br>-81.4 ± 15.3	R323, R288, K320, R348, R324, K393, K279, K287, R275, C389	2/1
		3	3	-120.8 ± 13.1<br>-102.0 ± 12.4<br>-112.9 ± 16.9	R323, R275, R288, R324, K287, K393, K271, R422, L273, R348	2/1
MODEL 2 HP, dp6	3, 2.95	1	6	-107.2 ± 11.2<br>-131.8 ± 9.7<br>-81.3 ± 8.3	K394, R348, K393, K295, K293, R324, M392, V347, M346, R422	3/3
		2	4	-84.3 ± 11.2<br>-60.7 ± 11.2<br>-72.0 ± 10.2	K295, R348, K394, K293, K393, K299, V347, K305, L296, V294	4/0
		3	4	-49.6 ± 15.0<br>-41.8 ± 16.8<br>-26.5 ± 9.2	K295, R288, K293, K344, K204, V294, R348, K299, K394, K287	4/0
MODEL 3 HP, dp6	2, 3.2	1	13	-6.6 ± 11.1<br>-34.9 ± 15.0<br>-41.6 ± 11.0	R275, K165, K271, K305, R288, K287, L273, Q403, P274, N407	7/6
		2	3	-37.5 ± 23.9<br>-41.4 ± 16.9<br>-50.2 ± 12.6	R275, K271, K165, R162, K287, K293, L273, K295, P274, R288	2/1
		3	3	-68.9 ± 12.6<br>-30.3 ± 19.3<br>-13.3 ± 17.5	K287, K271, K165, R288, K293, R275, K305, K295, K279, T290	2/1

DBSCAN parameters *m*, the minimal neighborhood size and ε, neighborhood search radius [33]; [2] Cluster number; [3] Cluster size; [4] Free energy of binding obtained by MM-GBSA; [5] Residues identified in the top 10 for binding according to MM-GBSA calculations per cluster ordered by the impact (starting from the most favourable one). [6] The polarity of a GAG binding pose was defined as its preferred orientation in relation to the reducing and non-reducing end.

Table S8. Fragment-based molecular docking MD analysis summary for PCPE-1 UNRES models/Ca^{2+}/HP dp11 interaction.

MODEL 1, HP dp11	1	–	-144.6±25.7	R323, R324, K287, R275, K394, R348, K393, K271, L273, R288
	2	–	-115.5±22.4	K393, R323, K394, R324, R275, K287, K305, R348, M392, R288
	3	–	-139.9±17.6	R323, R348, K279, K394, R324, R275, K393, R288, K344, R422
MODEL 2, HP dp11	1	–	-105.0±28.5	R422, K295, R288, K393, K293, K394, K344, R348, R324, K436
	2	–	-121.1±22.5	R422, K394, R324, K393, K293, K287, K434, R435, K295, K320
	3	–	-103.9±18.3	K394, K295, K293, K393, R422, R348, R324, K287, V294, R275
MODEL 3, HP dp11	1	–	-66.9±16.4	R422, K394, R324, R323, N424, K393, P423, Q427, K320, K387
	2	–	-111.4±19.1	K393, R422, K394, K344, R435, R348, V396, R324, G395, K436
	3	–	-90.9±26.9	R324, K393, K344, K394, R422, R348, R323, K320, M392, T325
MODEL 1, HP dp11	1	+	-157.1±18.3	R323, K287, K271, R275, K165, K320, R288, K393, R324, K394
	2	+	-151.4±24.5	R348, R324, R323, R275, K393, K394, K287, K320, K271, R288
	3	+	-110.4±16.6	R323, R324, R288, K436, K271, K287, K165, K320, K279, K299
MODEL 2, HP dp11	1	+	-135.3±33.6	R288, K394, K393, R422, R348, K436, R435, K287, K295, K299
	2	+	-130.7±26.3	K293, R422, R348, R324, K393, K295, K394, K287, R288, K299
	3	+	-141.2±21.7	R324, K394, K393, R422, R323, R348, K295, K299, K293, K287
MODEL 3, HP dp11	1	+	-52.1±26.8	K287, K299, K271, K279, R288, R275, K293, K295, R162, K165
	2	+	-99.0±33.0	K165, K271, R275, K295, K293, K287, P292, K204, R288, K279
	3	+	-69.7±13.8	R288, K287, R275, K293, K279, K295, T290, R323, K204, K165

[1] Pose number; [2] Ca^{2+} presence; [3] Free energy of binding obtained by MM-GBSA; [4] Residues identified in the top 10 for binding according to MM-GBSA calculations per cluster ordered by the impact (starting from the most favourable one).

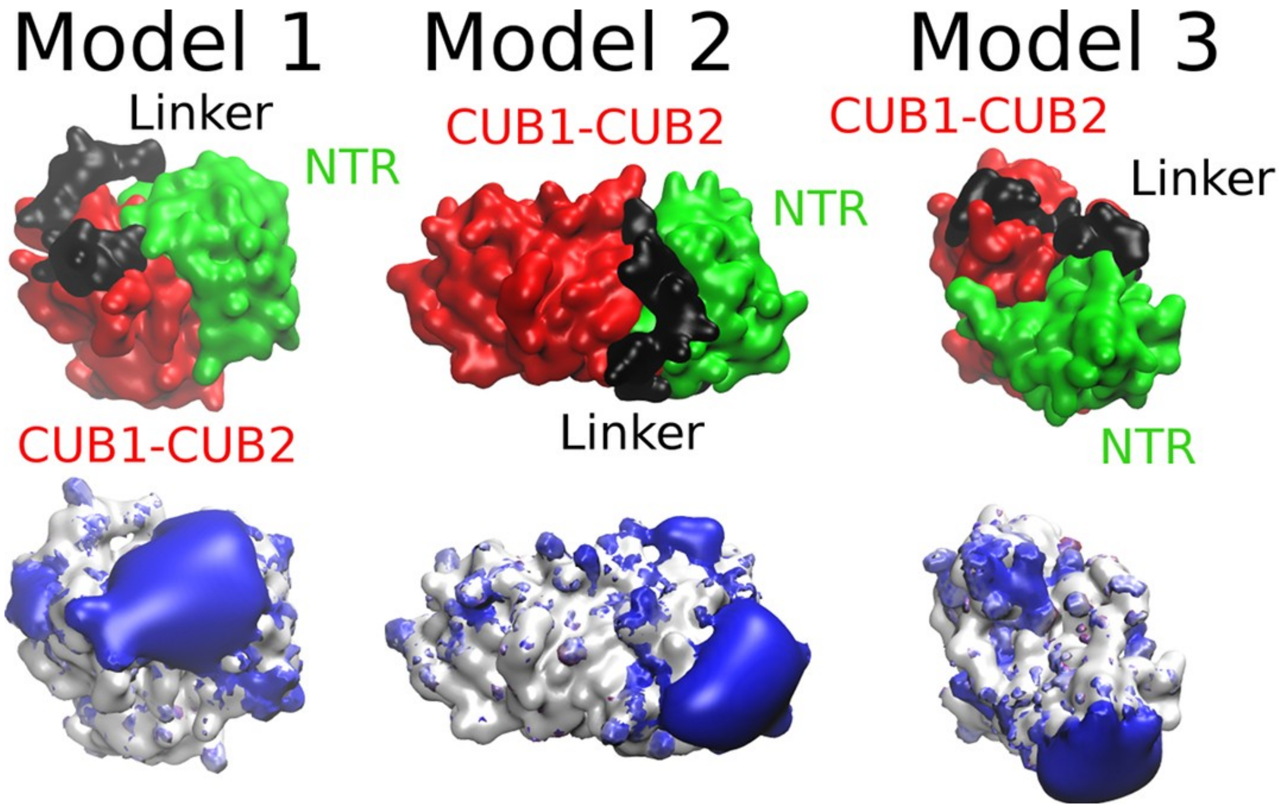

Figure S1. Models 1, 2, 3 (upper panel). NTR domain: green; CUB1-CUB2: red; the interdomain linker between the CUB2 and NTR domains: black. Positive electrostatic potential isosurfaces of PCPE-1 models (2.0 kcal/mol e^{-1}) in the absence of Ca^{2+} ions obtained by PBSA calculations.

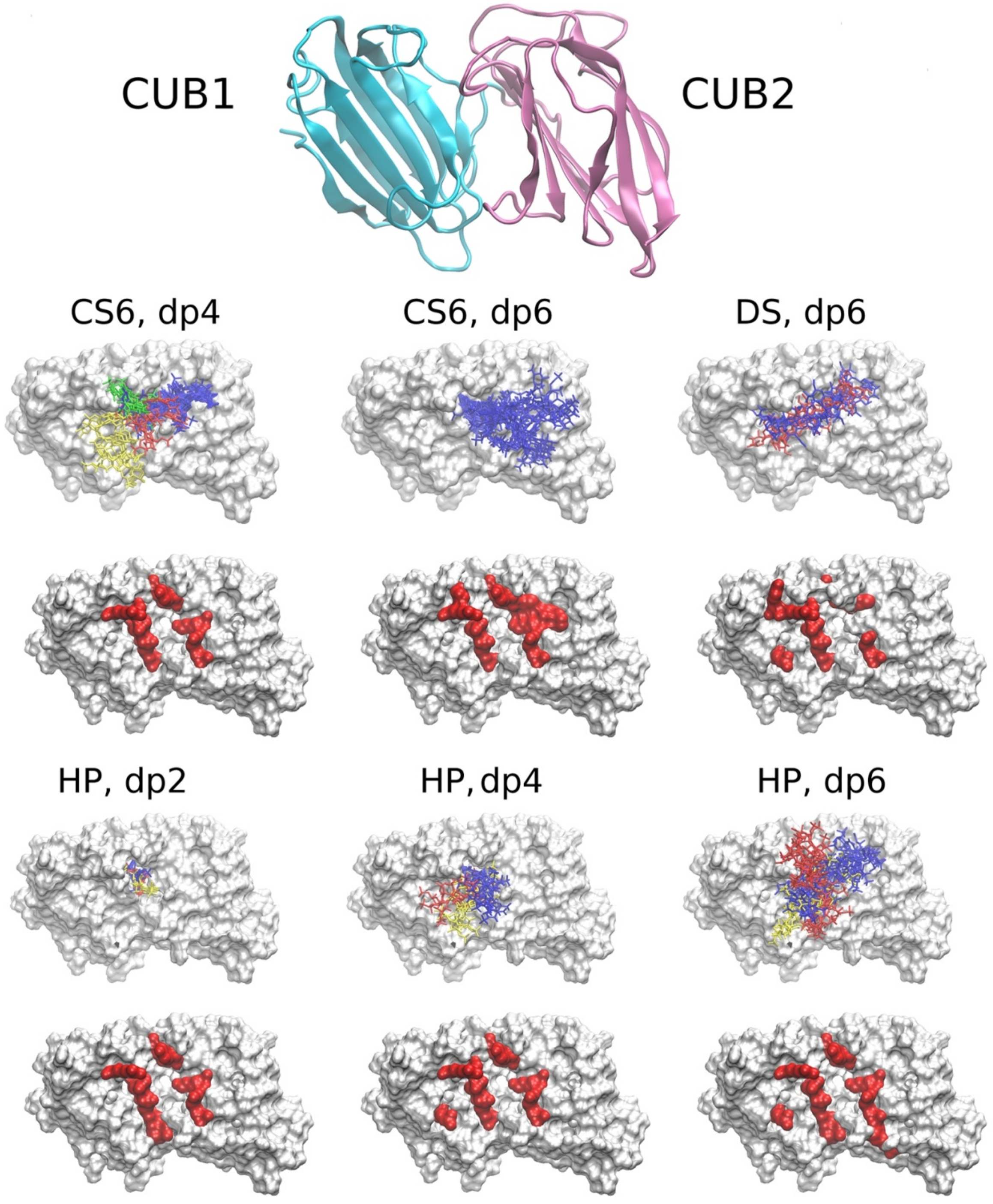

Figure S2. Molecular docking and MM-GBSA results for CUB1-CUB2-GAG complexes. The structure of the CUB1-CUB2 domains is shown in cartoon representation at the top. For each GAG, the analyzed clusters of docking solutions are shown in blue, red, yellow and green (from the most to the least populated cluster); the top 10 residues binding to GAGs according to MM-GBSA calculations averaged per GAG are highlighted in red surface, respectively.

```
  33      P V F L C G G D V K G E S G Y V A S E G F P N L Y P P N K E C I W T I T V P E G Q T V S L S F R V F 82
CS6 dp4
CS6 dp6
DS dp6
HP dp2
HP dp4
HP dp6

  83      D L E L H P A C R Y D A L E V F A G S G T S G Q R L G R F C G T F R P A P L V A P G N Q V T L R M T 132
CS6 dp4               *   *                               *   * * *
CS6 dp6           *       *                               *     *
DS dp6                *   * *                             *     *                                       *
HP dp2                    *                             * *   * * *
HP dp4                *   *                               *   * *                                       *
HP dp6                    *                             * *     *                                       *

 133      T D E G T G G R G F L L W Y S G R A T S G T E H Q F C G G R L E K A Q G T L T T P N W P E S D Y P P 182
CS6 dp4
CS6 dp6
DS dp6
HP dp2
HP dp4
HP dp6

 183      G I S C S W H I I A P P D Q V I A L T F E K F D L E P D T Y C R Y D S V S V F N G A V S D D S R R L 231
CS6 dp4                                                 *   *                                 *     *
CS6 dp6                                                 * * *                                 *     * *
DS dp6                                                    * *                                 *     *
HP dp2                                                  *   *                                 *     *
HP dp4                                                  *   *                                 *     *
HP dp6                                                  *   *                               * *     *

 232      G K G C G D A V P G S I S S E G N E L L V Q F V S D L S V T A D G F S A S Y K T L P R 275
CS6 dp4
CS6 dp6
DS dp6
HP dp2
HP dp4
HP dp6
```

Figure S3. CUB1-CUB2 amino acid residues identified in the top 10 for binding GAGs according to MM-GBSA calculations per cluster are labeled as an asterisk.

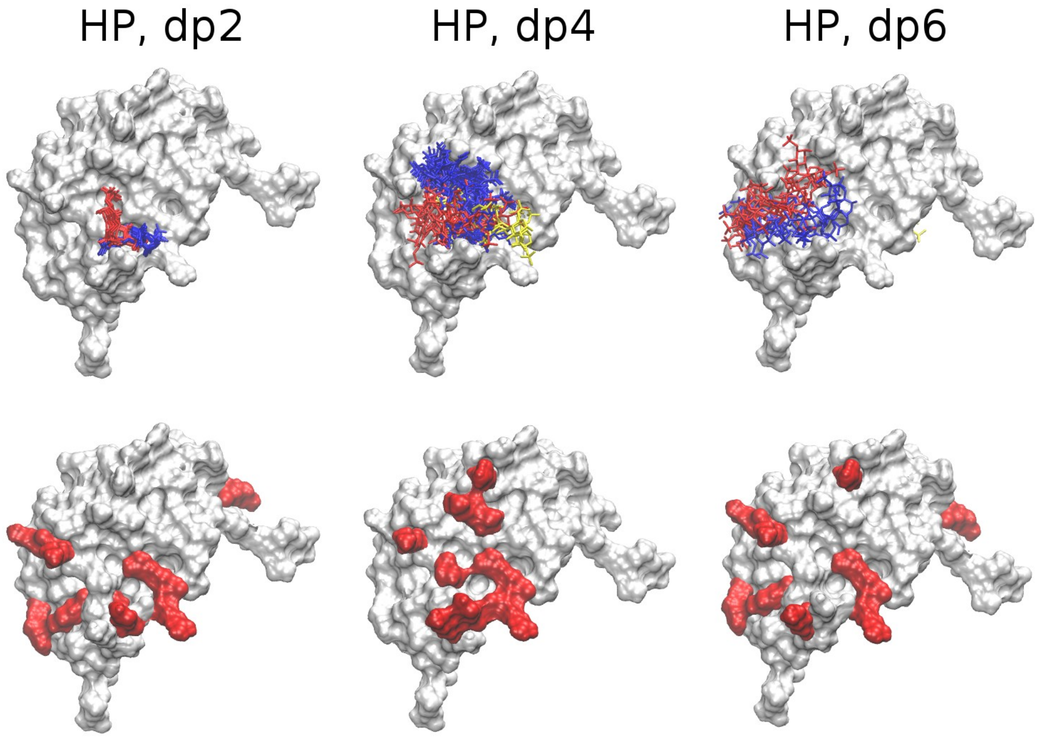

Figure S4. Molecular docking and MM-GBSA results for NTR/Ca^{2+}/HP systems. For each length of HP, the analyzed clusters of docking solutions are shown in blue, red and yellow (from the most to the least populated); the top 10 residues for binding GAGs according to MM-GBSA calculations averaged pro GAG are highlighted in red surface, respectively.

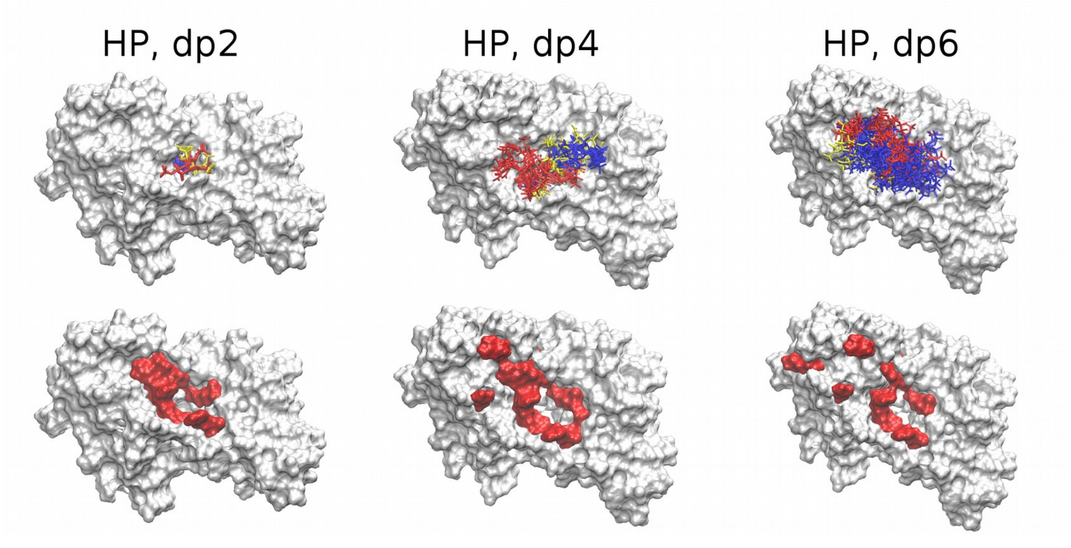

Figure S5. Molecular docking and MM-GBSA results for CUB1-CUB2/Ca^{2+}/HP systems. For each length of HP, the analyzed clusters of docking solutions are shown in blue, red and yellow (from the most to the least populated); the top 10 residues for binding GAGs according to MM-GBSA calculations averaged pro GAG are highlighted in red surface, respectively.

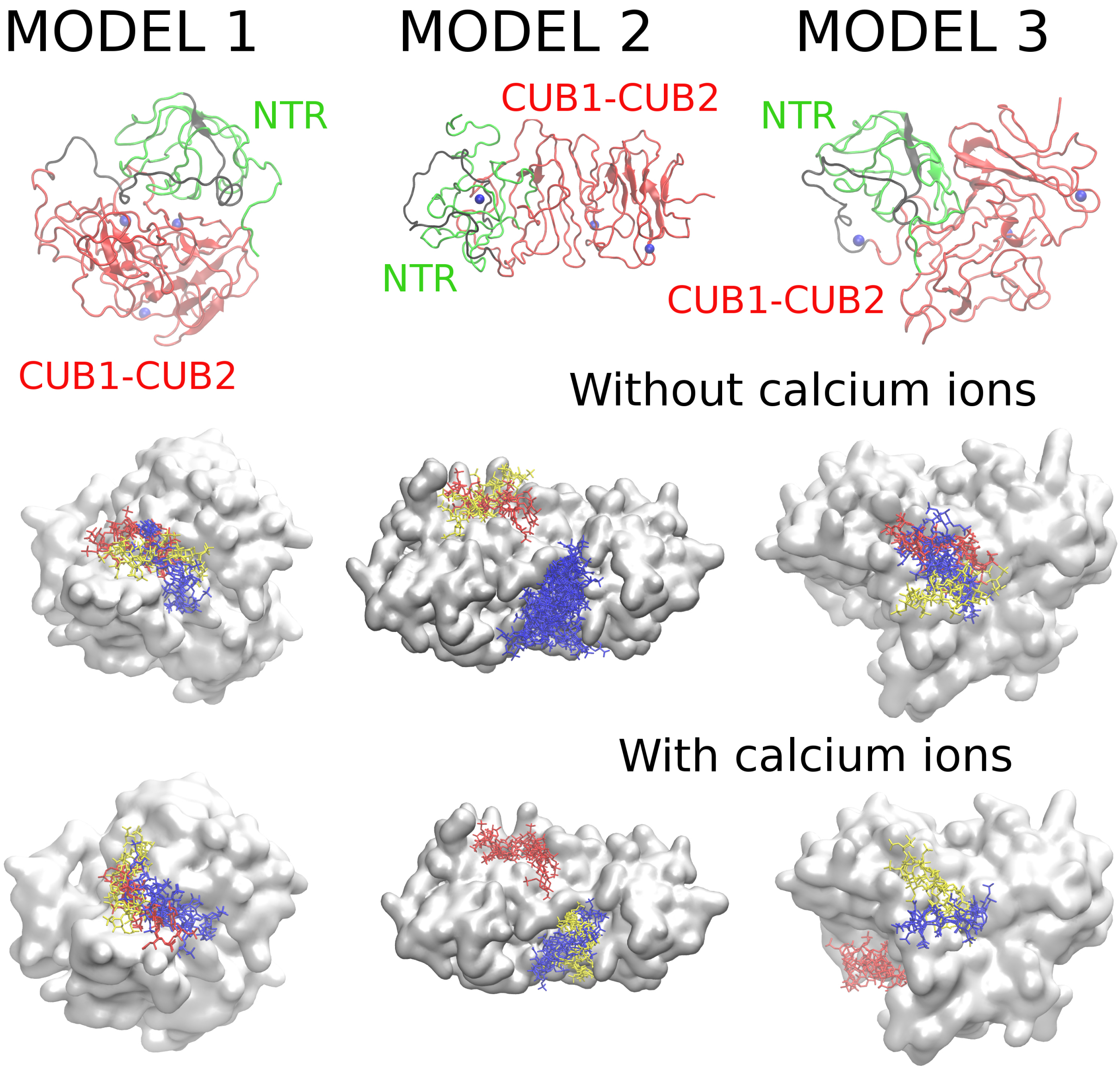

Figure S6. Molecular docking results for the models of full-length PCPE-1 protein in the absence and presence of Ca^{2+} ions and HP dp6. The clusters of docking solutions are shown in blue, red and yellow (from the most to the least populated clusters). NTR domain: green; CUB1-CUB2: red; the interdomain linker between the CUB2 and NTR domains: black.

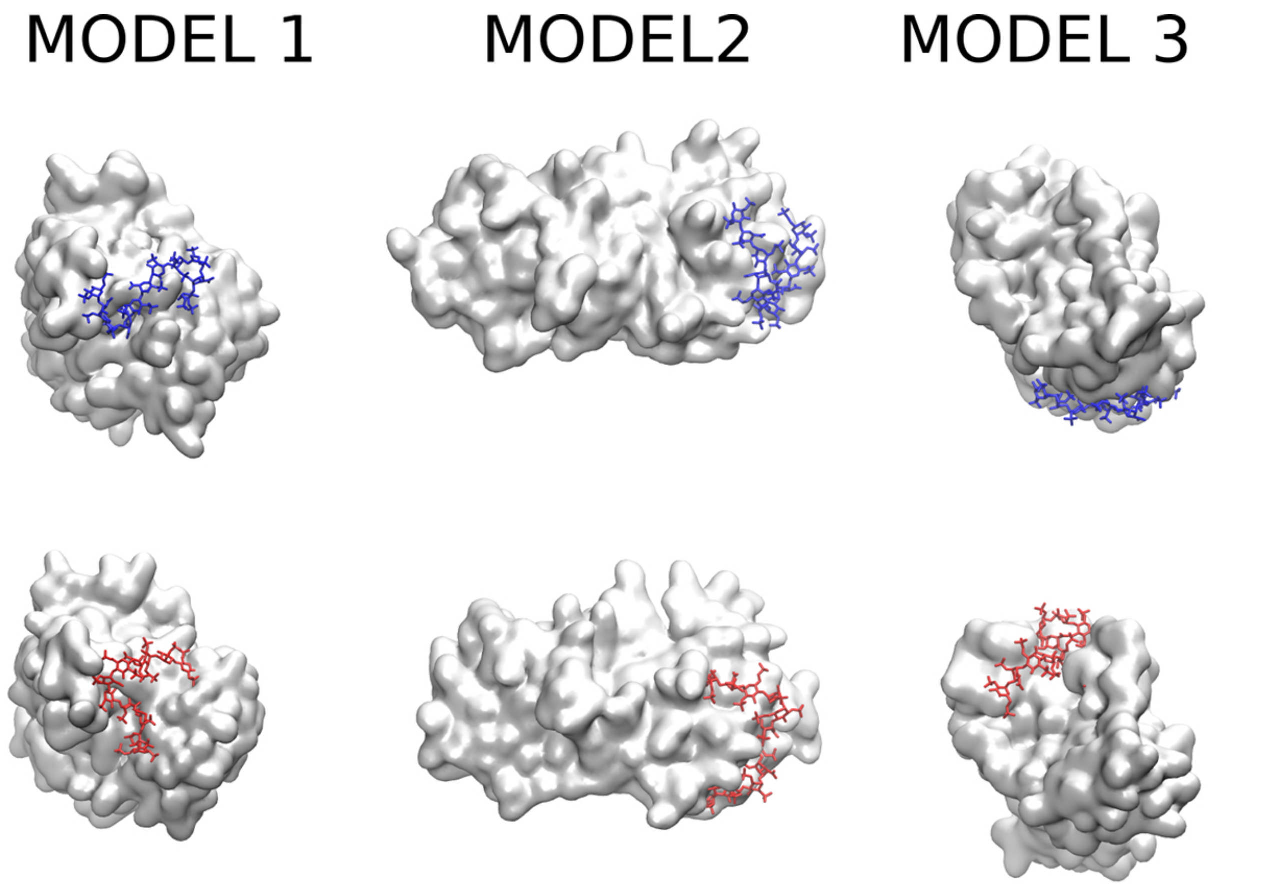

Figure S7. Molecular docking results for the models of full PCPE-1 protein in the absence (in blue) and presence (in red) of Ca^{2+} ions and HP dp11 corresponding to the most favourable free binding energies.
